# Being chair: a 12-step program for medical school chairs

**DOI:** 10.5116/ijme.4ece.862d

**Published:** 2011-12-01

**Authors:** Mark Fisher

**Affiliations:** University of California, Irvine, School of Medicine, Department of Neurology, USA

For a medical school-based career academician, the Department Chair position is a frequent, if not constant, focus of attention. There is an undeniable mystique about the position, and many academicians may find themselves wondering: “Maybe I could do this? Maybe I should do this? If it were me, I could do this so much better….”In some respects, the Chair is like any other or many other jobs. But the reality is that a medical school Department Chair is a unique position, with distinctive relationships and responsibilities. The scope of the position can be breathtaking, ranging from oversight of medical student training to maintenance of the highest quality patient care. Despite the importance of this position, there has been little attention in the literature to the realities of chairmanships and virtually nothing in terms of practical advice.For eight years I was a medical school Department Chair. When I look back I can only wonder how anybody manages to do the job. My formal preparation was basically nonexistent. There was, of course, on the job training, but there should be a better way.One way to approach a difficult situation requiring a new set of behaviors is a 12-step program. This approach has been widely utilized for alcoholism and drug addiction, but has also been incorporated in programs involving work behavior for psychiatrists and nurse practitioners.^1^^-^^3^ The principal value of 12-step programs relates to their simplicity and practicality.I have summarized my experience as Chair in the form of a 12-step program. I write this in the spirit of pointing out the pitfalls, landmines, and swamps that are going to be out there for all but the most fortunate Chairs. Let me offer some hard-earned and perhaps idiosyncratic advice. You can decide for yourself whether it qualifies as wisdom. This is what I wish someone had told me *before* starting as Chair.

## Case Report

I arrived my first day as Chair, at Grand Rounds, shortly after the beginning of the conference. My in-house rival for the job promptly stood up and left the room. It was the beginning of my real exposure to a Department in which (among other things) clinical programs were rudimentary or nonexistent, and the residency program was on probation and seemed ready to be disbanded. In fact, several years earlier there were serious discussions to merge the Department into another (and far better) Department at a nearby medical school. By the end of my Chairmanship, about thirty faculty members had been recruited, half a dozen clinical programs were established, the residency program was off probation and on solid footing, and the Department had achieved national recognition including entering the top ten in NIH research funding.

The 12 steps:

## 1. Welcome to middle management

The Chair position is truly middle management. You are the interface between faculty, residents, and staff (on the one hand) and the Dean’s Office (on the other). This is a middle management administrative position. That’s all it is. And, despite what you may have imagined, there is no throne or crown that comes with the position. You may wish to periodically remind yourself: middle management, administrative position. As mantras go, that one is not too bad for a Chair.

## 2. There is a learning curve for being Chair

This seems obvious, but will often be quickly forgotten. Chairs are chosen because they have been successful. They have run successful clinical programs, successful laboratories, successful program projects, successful residency programs. Success, success, success…. So, a Chairmanship is going to be just one more success story, right? Well, maybe…but it can also be an illustration of the Peter Principle, which, as you may recall is as follows: Everyone rises to their level of incompetence.The best way to avoid being a walking, talking illustration of the Peter Principle is to approach the position with a degree of humility. And humility may not be easy, given the past successes of the new Chair. But being Chair will be quite different from anything tackled before.The most important thing about the learning curve is to realize…there is a learning curve! The way you handle an issue when you start out as Chair will be different than how you would deal with it after being Chair for a number of years. On the job growth can occur, if you genuinely realize that you have a lot to learn.

## 3. Know your institution

Different institutions will have different attitudes and expectations for the Chair. That is pretty obvious. What is less obvious is that different classes of institutions will view Chairs differently. Specifically, the differences between a public and private institution have not been that well appreciated, at least as they pertain to the Chair.

Chair authority at private institutions tends to be far greater than at public institutions. Accountability to faculty will be substantially more at public institutions, where a Chair can easily feel squeezed between the Dean’s expectations and the demands of the faculty. Private institution Chairs can be more dictatorial, I mean authoritarian, and get away with it. Formal Chair reviews are common in public institutions, but may be absent at private institutions.

The private/public distinction for Chair authority is particularly important at the beginning of the Chairmanship. Chairs are often recruited to and enter an institution as Department Chair. The transition from being a faculty member at a private institution to being Chair at a public institution will create (at the very least) some significant culture shock.

## 4. Getting to know your predecessor

More likely than not, your predecessor Chair will remain in the Department as a faculty member. And not just as any faculty member, but as the person who has been through it and seen just about all of it before. Some of the faculty will still see your predecessor as the true leader of the Department, title or no title.Your predecessor will view you with some serious ambivalence. Your successes will not generate wholehearted admiration, and your failures may not create much sympathy. Your predecessor will be intently interested in what is happening in the Department, but is likely to be at least as interested in their own Chair legacy. Unfortunately, the reputation of your predecessor may be enhanced by your difficulties, and your successes may even be viewed as diminishing your predecessor’s legacy.Given these murky attitudes, what is the best approach? That’s easy. Treat your predecessor with due respect. Set up meetings, lunches or breakfasts, to catch up. Let your predecessor mentor you. Nothing is more flattering to your predecessor than the belief that you want their mentoring. The good news is: You will really benefit from what your predecessor has to say. And the act of mentoring will engage your predecessor and give a stake in your success. And you will have an ally, which is always a good thing.

## 5. Understand how you will be evaluated

You have a good idea of what you want to accomplish as Chair. You have seen the workings of a Department for quite a while now, and you know what needs to be done. So, you set about accomplishing this, achieve it, and voila, you are a successful Chair! Right? Well, not necessarily.

As Chair, you will be reviewed. It may be a formal review, it may be an informal review. But your performance will be reviewed. And the criteria of this Chair review may have absolutely nothing to do with what you thought was important.

So, one of the first things to do is to find out exactly how the review process will unfold. What are the institution’s criteria for a successful Chair? The review criteria may well be available on paper, for your own perusal. Or it may require a detailed discussion with the Dean.

But be very clear on this: You will need to come to grips with your institution’s review criteria, even if they are vastly different from what you think they should be. What you want to avoid is a situation where by your own criteria you are doing a great job, but by the institution’s criteria and expectations, you are not so hot.

The tough part may come when you realize that what you think is important for the Chair, what you want to accomplish as Chair, may simply not be what the institution had in mind. You will then be left with adjusting your expectations to the realities of your institution. Either that, or you will be left with a very unhappy Chair experience.

## 6. Know who your role models really are

You have role models for the Chair, of course. There are Chairs out there who you admire, respect, and want to imitate. But are they the ones who are really your role models?

It is entirely possible, even likely, that you don’t know who your real role models are. Here’s a clue: think about your own Department Chair. Whether you like it or not, whether you are aware of it or not, you are likely to be re-enacting your former Chair(s), at least some of the time.

I once joked (sort of) that when I was faced with a difficult problem, I would ask myself “what would (my former Chair) do in this situation?” And then I thought it through, carried it out, and when I did it…it was a disaster!

Of course it didn’t work, because of the vastly different personalities and circumstances. But the point here is this: You may or may not realize it, you may not like it, but you are going to be viewing events through the prism of your experience and relationship with your former Chair. At least, you will at the onset of your Chairmanship. So the quicker you realize this, the better off you will be.

## 7. Staff and a Chair’s success

Your attention will no doubt focus on your faculty, your residents, and medical students. That’s fine. But you will probably overlook the most easily overlooked members of your Department: your staff.

Staff can make a Department or Chair look good, or look terrible. As Chair, you will be spending more time with staff than you ever have previously. And one of your biggest roles is to be supportive of your staff. That may not sound too exciting, and yet it can be the most satisfying part of your job as Chair. That’s right, better than Department rankings and recognition and all that other stuff. You will have a degree of emotional intimacy (and I mean that in a good way) with your staff unlike any other relations in the Department. So make the most of it, and you will be amazed how much you and the Department will benefit.

## 8. Alienation is easy, reconciliation is hard

You are used to having casual conversation with colleagues in your Department, of course. You engage in casual conversations and make casual comments. But now you are Chair, and you will find that some casual comments aren’t so casual anymore. Your words are taken far more seriously. Your words are repeated, sometimes mistakenly, and sometime even distorted.

Academic physicians tend to have pretty fragile egos. Motivation for many physicians to enter academia is at least partially related to a desire for recognition, maybe to assuage that fragile ego. This is part of what has been termed a narcissistic personality, which is an interesting topic in itself for academicians. But the larger and more immediate issue for the Chair is this: It doesn’t take much to damage that fragile ego of your Department faculty member. And a thoughtless comment coming from the Chair is doubly damaging (and infuriating).

Narcissistic injury generates narcissistic rage. In other words, it is easy for a Chair to make an enemy. And that enemy will return the favor of narcissistic injury, this time directed back at the Chair. And so an academic feud is born, a feud arising out of who knows what. And resolving that feud will take motivation and understanding that is beyond the skill set of most academicians.

## 9. You can never have too many meetings

One of your first (and often overlooked) tasks as Chair is to establish a certain climate. Actually, you will be establishing a climate, whether you intend to or not. Ideally, that climate will encompass collegiality, respect, and integrity. Easier said than done….The best way to establish the right climate is by meetings. And this is what being Chair is all about: meetings, meetings, and more meetings. Not emails…meetings!I know, I know…meetings are boring! Of course they are. And here’s the secret: They are supposed to be boring!That’s right, the meetings are supposed to be boring, or at least most of the meetings are supposed to be boring. The fact that meetings are boring (and I am primarily talking about meetings with faculty) is actually a good sign. It means that the important issues are being addressed, and that there is little excitement left in issues.On the other hand, if the meetings are exciting, well, you are in trouble! You need to have enough meetings to digest the tough issues to a sufficient extent that any significant excitement is largely drained out of them.So, have your meetings, and expect them to be boring. And to get there, you can never have too many meetings.

## 10. The key to recruitment

Probably the most exciting thing about being Chair is recruiting. This is the real building part of the job. New people, new energy, new ideas…and maybe new problems.What are you looking for in that recruit? Raw academic talent? An academic pedigree that will look good (on paper at least) for the Department? Energy and ambition?Probably the best way to think about it is to view this as if you are bringing someone into your family. An academic department can create extraordinary closeness, connections, emotions.Your emotional reaction to the recruit will tell the story. Do you like this person or not? Can you trust them? Would you want to be in the Department with them if you weren’t Chair?Far too many talented recruits turn out to be world-class headaches. In administrative jargon, they are “high maintenance” faculty. They don’t need a Chair, they need treatment. When recruiting, trust your gut.

## 11. When it’s time to go

One of my predecessor Chairs is fond of zen-like aphorisms, and one of his favorites is that the Chair “is not a life sentence”. You may have started as Chair with a predetermined limited time frame for the position. But the perks of the position, the sense of entitlement, the stipend, and the (superficial) deference can prove to be addictive. What seemed like a few years becomes, well, a life sentence.

But the reality is that only you know if you are enjoying the experience. Excitement may be replaced by dread and boredom. Your mind wanders incessantly during the meetings. You have mastered the job, but wonder if Chair mastery really means all that much. Your relations with the Dean have changed, and maybe there is a new Dean in place. The rather benign father figure at the onset has become an increasingly difficult taskmaster. Your meetings with the Dean have an edge, and are sometimes adversarial.

There are, of course, a wide variety of variations on this theme. But regardless of the circumstances, it is essential to remember that you are working at the pleasure of the Dean. And if the Dean really is no longer pleased, it’s over.

## 12. Life post-Chair

I became convinced some time ago that the person who becomes Chair will experience a rapid decline in intelligence, at least a 10% drop of IQ (I know I certainly did). Why do I say such a thing? That’s easy: just look at all the dumb things Chairs do!The good news is, this intellectual decline is reversible…so, enjoy your rebound!

## Discussion

The 12-step program described herein provides a unique and practical approach to issues a Chair must face. While casual if not irreverent at times, it has the advantage of accessibility and easy engagement. The question remains, however, how this approach may compare to current and traditional views of the Chair’s position. The need for substantial input to new Chairs is clearly recognized.^4^ The standard and most comprehensive work on Department Chairs is Biebuyck and Mallon’s three volume “The Successful Medical School Department Chair: A Guide to Good Institutional Practice”.^5^^-^^7^ This is a remarkable work, covering a wide range of topics. However, at nearly 300 pages spanning three volumes (or “modules”), it is somewhat intimidating in size. Moreover, it is written principally for the edification of, and as a reference for, medical school Deans, rather than Department Chairs. Probably the most relevant section for Chairs is in the fourth chapter of the second volume, titled “Skill Sets for the Department Chair”.^6^ This chapter reviews some “soft-side” skills, specifically “managing conflict”, “performance evaluation”, and “managing diversity”. These brief discussions provide some helpful insights and offer suggestions on resources available to help a Chair. Similar works offering advice to medical school Deans are also available on a much smaller scale.^8^^-^^11^ Interestingly, “trust” was recognized as the most important of all leadership values for a Department Chair.^11^Trends in Chair recruitment and retention are well described in the literature. Average tenure for a Chair (during the 1990’s) was eight years, a reduction of approximately 20% from a prior survey period for the early 1980’s.^12^Increased turnover rates among Chairs of several specialties have also been reported.^13^^,^^14^ Supplementing these observations is the emerging literature on Chair “burnout”.^15^^-^^17^ Burnout is now well-defined as a syndrome of emotional exhaustion (i.e. feeling overextended and exhausted by work), low sense of personal accomplishment, and depersonalization (i.e. callous and dehumanized treatment of others).^15^ Burnout has been distinguished from depression in that the former is specific for the workplace, rather than involving both personal and professional life.^17^ Moderate or high burnout rates of up to 84% of Department Chairs have been reported, with the best predictors of burnout being low sense of self-efficacy (i.e. effectiveness), poor spousal support, disputes with the Dean, department budgetary difficulties, and working nights and weekends.^15^ While these observations may provide limited guidance for Chair behavior, they do point to frequently low job satisfaction and the need for improving the Chair experience.Other characteristics of a successful Department Chair have received some attention. The Chair as “middle management” is described in at least one other publication.^18^ The importance of the Chair being a “role model”, a “coordinator” and manager of change for faculty and residents has also been described.^19^^-^^21^ The difficulties a Chair may encounter as a researcher have also received attention^22^, along with potential gender-dependent differences in Chairs.^23^The literature on medical school Department Chairs is thus focused primarily on advice to medical school Deans, along with trends in Chair tenure and burnout. While characteristics of successful Chairs are described, direct advice is generally lacking. The current proposed 12-step model for Chairs thus seems consistent with and a useful supplement to the existing Chair literature.

### Conflict of Interest

The author declares that he has no conflict of interest.
